# Association Between ω‐3, ω‐6 Polyunsaturated Fatty Acid and Sleep Disorders: From Cross‐Sectional to Mendelian Randomization Studies

**DOI:** 10.1002/fsn3.70311

**Published:** 2025-05-27

**Authors:** Lin Wang, Wei Quan, Jia Song, Yidan Qin, Huibin Zeng, Jian Zhang, Xuan Zhao, Jia Li, Jiajun Chen

**Affiliations:** ^1^ Department of Neurology China‐Japan Union Hospital of Jilin University Changchun China

**Keywords:** mendelian randomization, NHANES, omega‐3, omega‐6, polyunsaturated fatty acids, sleep disorders

## Abstract

Sleep disorders are a common health problem affecting a significant proportion of the adult population. Emerging evidence suggests that dietary factors, particularly polyunsaturated fatty acid (PUFA) intake, may play a role in modulating sleep quality. This study aims to investigate the association between omega‐3 (ω‐3) and omega‐6 (ω‐6) PUFA and sleep disorders using cross‐sectional survey data and data from genome‐wide association studies (GWAS). Using data from the National Health and Nutrition Examination Survey (NHANES, 2005–2018), we analyzed a cohort of 31,920 participants, with the primary independent variables being intake of ω‐3 and ω‐6 PUFAs. Multivariate regression was used to assess associations, and restricted cubic spline analysis was used to explore potential non‐linear dose–response relationships. Two‐sample Mendelian randomization (MR) analyses were performed to evaluate the causal effects of levels of multiple fatty acids on the risk of sleep disorders. For analysis on NHANES data, the participants with sleep disorders had significantly lower ω‐3 PUFA intake (1.71 ± 1.11 g) compared to those without sleep disorders (1.78 ± 1.14 g, *p* < 0.001). Regression analysis revealed that higher ω‐3 PUFA intake was associated with a reduced risk of sleep disorders, while the MR analyses showed that a higher ratio of ω‐6 to total fatty acid levels was causally associated with a lower risk of sleep disorders (IVW OR = 0.930, 95% CI: 0.880–0.983, *p* = 0.011). Our findings suggest that increased ω‐3 FA intake and increased ratio of ω‐6 to total fatty acid level may be associated with a lower risk of sleep disorders, highlighting the potential benefits of dietary modification for sleep health. Future research should further explore these associations and consider intervention studies to establish causality and optimal dietary recommendations to prevent sleep disorders.

## Graphical abstract

By integrating National Health and Nutrition Examination Survey (NHANES, 2005–2018) data analysis with Mendelian randomization analysis, we demonstrated that the increased ω‐3 FA intake and increased ratio of ω‐6 to total fatty acid level may be associated with a lower risk of sleep disorders, highlighting the potential benefits of dietary modification for sleep health.

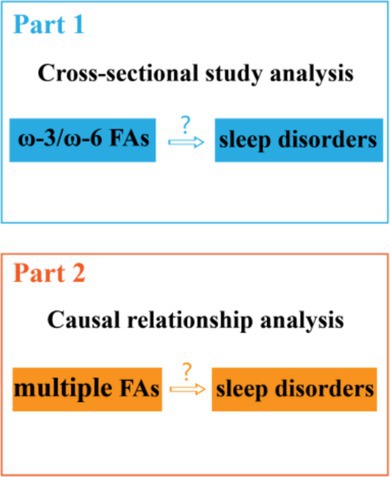

## 1 Introduction

Sleep disorders are increasingly being acknowledged as a significant public health concern, affecting millions of individuals across the globe. In the United States alone, it is estimated that approximately 50–70 million adults experience some form of sleep disorder (Shokoueinejad et al. 2017). These disorders are associated with various adverse health outcomes, including cardiovascular diseases, diabetes, and impaired cognitive function (Miller and Howarth 2023; Reutrakul and Van Cauter 2018; Yaffe et al. 2014). Despite the availability of diagnostic tools and treatments such as pharmacological treatments and behavioral interventions, many sleep disorders remain underdiagnosed and undertreated (Xu et al. 2022; Jaqua et al. 2023). This highlights the necessity for further research into the factors influencing sleep health and the development of more effective intervention strategies.

Polyunsaturated fatty acids (PUFAs), particularly omega‐3 (ω‐3) and omega‐6 (ω‐6) fatty acids, have been the subject of considerable interest with regard to their potential role in the modulation of sleep health. ω‐3 PUFAs, which are commonly found in fish oil, have anti‐inflammatory properties and are essential for brain health (Laye et al. 2018). On the other hand, ω‐6 PUFAs, which are found in vegetable oils, have been identified as precursors to pro‐inflammatory molecules (Yan et al. 2023). It is thought that the balance between these fatty acids exerts an influence on a number of physiological processes, including sleep. However, the relationship between ω‐3 and ω‐6 and sleep disorders is controversial. A series of studies indicated that the ω‐3 FA level was positively associated with sleep quality (Montgomery et al. 2014; Del Brutto et al. 2016; Irmisch et al. 2007; Scorza et al. 2013; Tang et al. 2018). There is some evidence that ω‐6 FA has a different role than ω‐3 FA in sleep. For example, a cross‐sectional study using the data of NHANES 2007–2016 showed that ω‐6 consumption and the ratio of ω‐6 to ω‐3 were positively associated with the risk of sleep disorders (Luo et al. 2021). A clinical trial demonstrated that the sleep quality in migraine sufferers can be significantly improved by increasing ω‐3 FA in the diet, with or without reducing ω‐6 FA (Faurot et al. 2023). However, many reports have failed to find a positive association between ω‐3 FA and sleep quality (Hysing et al. 2018; Cornu et al. 2010; Cohen et al. 2014; Hansen et al. 2014). Some studies even reach the opposite conclusion: for example, a meta‐analysis by Rachel A. Murphy et al. found that long‐chain ω‐3 FA concentration was negatively associated with sleep duration (Murphy et al. 2022). In addition, the relationship between ω‐6 FA and sleep remains uncertain. In a cross‐sectional study of Chinese children and adolescents, no significant association was found between erythrocyte membrane ω‐6 FA levels and sleep disorders (Tang et al. 2018). Nevertheless, there is currently no conclusive evidence for these relationships, and further research is needed to clarify them.

The National Health and Nutrition Examination Survey (NHANES) provides a valuable dataset for investigating the associations between dietary intake and health outcomes in a representative sample of the U.S. population. However, studies based on NHANES data are cross‐sectional and have the following limitations: First, it is impossible to determine the causal relationship between exposures and diseases. Therefore, it can only show associations and not establish causal relationships. Second, it can only reflect the prevalence of disease at a given point in time and cannot provide information on morbidity. Finally, the accuracy of the data is affected by recall bias. Mendelian randomization (MR) analysis can address some of the limitations of cross‐sectional studies. Firstly, as genetic variation is independent of environmental and lifestyle factors, it can effectively control confounding variables that may influence health outcomes, thereby enhancing the objectivity of the research findings. Secondly, by employing genetic variation as a tool variable, it is possible to more accurately identify true causal relationships, rather than merely correlational ones. Furthermore, genetic variation is fixed at the time of conception, eliminating the issue of reverse causality.

In this study, we utilized data from the NHANES database, focusing on 31,920 participants aged 20 years and older. Sleep disorders were assessed through self‐reported questionnaires, categorizing participants into groups with and without sleep disorders. Dietary intake of ω‐3 and ω‐6 PUFAs was calculated based on the average of two 24‐h dietary recalls. Adjusted multivariable logistic regression models were employed to evaluate the associations, controlling for potential confounders such as age, sex, race, education level, marital status, family income, body mass index (BMI), physical activity, smoking status, and comorbidities like diabetes and hypertension. Additionally, restricted cubic spline analyses were performed to explore potential nonlinear dose–response relationships. Then a two‐sample MR analysis was applied to evaluate whether there were causal effects of multiple circulating fatty acids (omega‐3 FA, omega‐6 FA, omega‐6/omega‐3, omega‐3/total FA, omega‐6/total FA, docosahexaenoic acid, docosahexaenoic acid/total FA, linoleic acid, linoleic acid/total FA, monounsaturated FA, polyunsaturated FA, saturated FA, and total FA) on the risk of sleep disorders.

This study aims to fill this gap by examining the association between ω‐3 and ω‐6 FA and sleep disorders using NHANES data (2005–2018) and MR analysis. By identifying dietary factors that influence sleep health, this research could inform public health guidelines and contribute to the development of targeted nutritional interventions aimed at improving sleep quality and overall health.

## 2 Methods

### 2.1 Study Design and Data Availability

**FIGURE 1:**
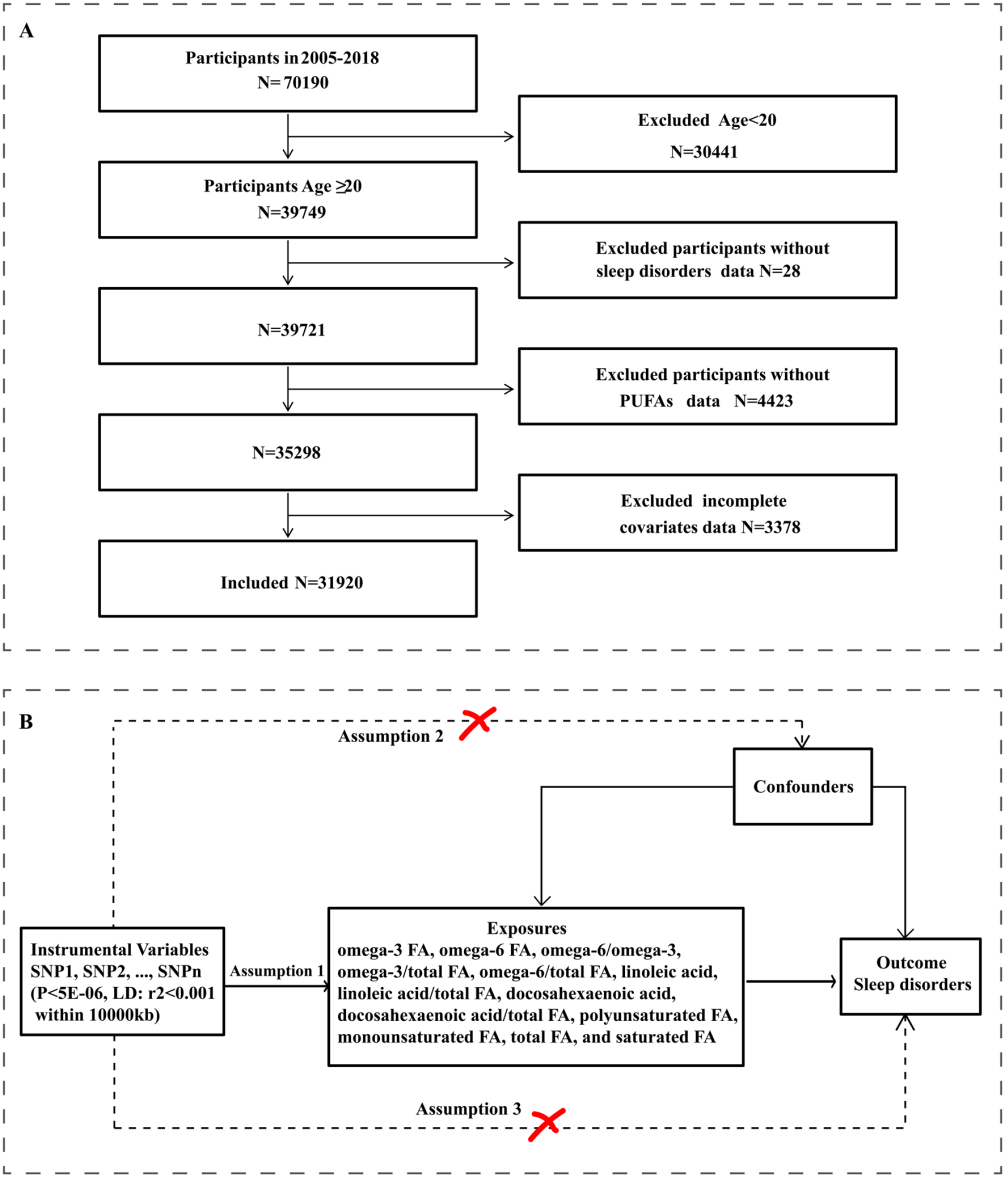
Flowchart for this study. (A) Flowchart of participant selection from NHANSE database. (B) Flowchart of the MR analysis. FA, fatty acids; LD, linkage disequilibrium; SNP, single nucleotide polymorphism.

In this study, data from NHANSE (2005–2018) was used to obtain 70,190 participants. Firstly, 30,441 participants under the age of 20 were excluded. Secondly, 28 participants without sleep disorders were excluded. Then 4423 participants without PUFA data were excluded. Finally, 3378 participants with incomplete covariates were removed, leaving 31,920 participants included in the study. The process of sample selection was illustrated in Figure 1A.

Then a two‐sample MR analysis was performed to determine the causal impact of multiple circulating fatty acids on the risk of sleep disorders Figure 1B. The selection of instrumental variables (IVs) was guided by the following three basic assumptions: (1) the selected IVs must be significantly associated with the exposure (*p* < 5E‐06); (2) the IVs are independent from confounders that may influence the exposure and outcome; (3) the IVs can only influence the outcome through their effects on the exposure and cannot influence the outcome by other means (LD: *r* ^2^ < 0.001within 10,000 kb). MR analysis in this study adhered to STROBE‐MR guidelines (Skrivankova et al. 2021). A STROBE‐MR checklist was shown in Table S1.

The GWAS data used for MR analysis were obtained from publicly available GWAS databases, and detailed information was provided in Table 1. The datasets for fatty‐acid‐related traits, including omega‐3 FA, omega‐6 FA, omega‐6/omega‐3, omega‐3/total FA, omega‐6/total FA, docosahexaenoic acid, docosahexaenoic acid/total FA, linoleic acid, linoleic acid/total FA, monounsaturated FA, polyunsaturated FA, saturated FA, and total FA, were obtained from the MRCIEU GWAS (https://gwas.mrcieu.ac.uk/) (Richardson et al. 2022). The outcome dataset of sleep disorders was obtained from the FinnGenR10 with 49,880 cases and 358,194 controls (https://storage.googleapis.com/finngen‐public‐data‐r10/summary_stats/finngen_R10_SLEEP.gz) (Kurki et al. 2023). Missing data was deleted. The exposure and outcome data above are from European populations. The protocol and details of the GWAS studies included in this study were pre‐registered (accession IDs GCST90092816, GCST90092817, GCST90092880, GCST90092881, GCST90092928, GCST90092931‐GCST90092935, GCST90092939, GCST90092980, GCST90092987, https://www.ebi.ac.uk/gwas/).

**TABLE 1:** GWAS data used in this study.

Exposure	GWAS ID	Sample size	SNPs	Population	PMID
Omega‐3 FA	ebi‐a‐GCST90092931	1,15,006	1,15,90,399	European	35,213,538
Omega‐6 FA	ebi‐a‐GCST90092933				
Omega‐6/Omega‐3	ebi‐a‐GCST90092934				
Omega‐3/Total FA	ebi‐a‐GCST90092932				
Omega‐6/Total FA	ebi‐a‐GCST90092935				
Docosahexaenoic acid	ebi‐a‐GCST90092816				
Docosahexaenoic acid/Total FA	ebi‐a‐GCST90092817				
Linoleic acid	ebi‐a‐GCST90092880				
Linoleic acid/Total FA	ebi‐a‐GCST90092881				
Monounsaturated FA	ebi‐a‐GCST90092928				
Polyunsaturated FA	ebi‐a‐GCST90092939				
Saturated FA	ebi‐a‐GCST90092980				
Total FA	ebi‐a‐GCST90092987				

Outcome	GWAS ID	nCases	nControl	Population	Consortium
Sleep disorders	finngen_R10_SLEEP	49,880	3,58,194	European	FinnGen

### 2.2 Variables

For the analysis on NHANSE data, the outcome variables included were patient self‐reported data on sleep disorders and in the questionnaire data, participants were asked “Have you ever told a doctor or other health professional that you have trouble sleeping? Participants were included in the sleep disorder group if they answered ‘yes’ and in the no sleep disorder group if they answered ‘no’” (Zhong et al. 2024).

The independent variables included were ω‐3 and ω‐6 FA intake. ω‐3 FAs include α‐linoleic acid (ALA, 18:3), docosapentaenoic acid (DPA, 22:5), docosahexaenoic acid (DHA, 22:6), eicosapentaenoic acid (EPA, 20:5) and stearidonic acid (SDA, 18:4). ω‐6 FAs include eicosatetraenoic acid (AA, 20:4) and linoleic acid (LA, 18:2) (Li et al. 2024). In addition, the sum of ω‐3 FA intake and ω‐6 FA intake and the ratio of ω‐3 FA intake to ω‐6 FA intake were also included in this study. FA intake data were derived from the mean of two 24‐h dietary recalls from the NHANES database, with the first 24‐h dietary recall conducted at the Mobile Examination Centre (MEC) and the second 24‐h dietary recall conducted by telephone interview.

Covariates included: age, sex, race, educational level (Edu), marriage, PIR, BMI, physical activity status (Activity), smoking, and comorbidities (diabetes and hypertension). Age grouping: ≤ 60 years, > 60 years; race grouping: non‐Hispanic white, non‐Hispanic black, Mexican American, or other races; smoking grouping: never (< 100 lifetime cigarettes), former (> 100 lifetime cigarettes but not a current smoker), or current; activity grouping: yes (moderate or vigorous activity), no (no moderate or vigorous activity).

### 2.3 Heterogeneity and Pleiotropy Tests

Cochran's *Q*‐statistic was used for the heterogeneity test, and the *Q*_pvals larger than 0.05 were considered no heterogeneity. The MR‐Egger intercept method was performed for the horizontal pleiotropy test, and the samples with *p* < 0.05 were excluded from the subsequent analyses. In addition, the effect of removing each SNP on the remaining SNPS was assessed using the leave‐one‐out method.

### 2.4 Statistical Analysis

A multivariate logistic regression model was used to assess the association between omega‐3 and omega‐6 FA intake and sleep disorders by odds ratio (OR) and 95% confidence interval (95% CI). Model 1 was adjusted for none; model 2 was adjusted for age, sex, race, Edu, marriage, PIR, and BIM; model 3 was adjusted for age, sex, race, Edu, marriage, PIR, BIM, activity, smoking, diabetes, and hypertension. Restricted cubic spline (RCS) plots were used to analyze possible non‐linear dose–response relationships between ω‐3 and ω‐6 FA intake and sleep disorders. In addition, subgroup analyses were conducted to test the stability of the relationship between ω‐3 and ω‐6 FA intake and sleep disorders. All IVW results were corrected by multiple tests using the FDR method.

Statistical analyses were performed using R software (version 4.3.3), and *p* < 0.05 was considered statistically significant. R packages “MRPRESSO” (Verbanck et al. 2018), “TwoSampleMR” (Rasooly and Patel 2019), and “MendelianRandomization” (Yavorska and Burgess 2017) were used for MR analysis. Continuous variables were expressed as mean ± standard deviation, and one‐way ANOVA was used for comparisons between groups. Data for categorical variables were expressed as number of cases (%) and comparisons between groups were made using the chi‐square test.

## 3 Results

### 3.1 Characteristic Analysis

Of the 31,920 people included in the study, 8225 (26%) were in the sleep disorder group and 23,695 (74%) were in the no sleep disorder group. The baseline characteristics of all participants included in the study were shown in Table 2. The mean ± variance of ω‐3 FA intake among all participants included in the study was 1.76 ± 1.13, with 1.71 ± 1.11 in the sleep disorder group and 1.78 ± 1.14 in the no sleep disorder group. ω‐6 FA intake had a mean ± variance of 15.78 ± 9.26, with 15.89 ± 9.28 in the sleep disorder group and 15.48 ± 9.18 in the no sleep disorder group. The mean ± variance of the sum of ω‐3 FA intake and ω‐6 FA intake was 17.55 ± 10.19, of which 17.19 ± 10.10 in the sleep disorder group and 17.67 ± 10.10 in the no sleep disorder group was 17.67 ± 10.22.

**TABLE 2:** Baseline characteristics of the included participants.

Characteristic	Overall, *N* = 31,920	No sleep disorder *N* = 23,695 (74%)	Sleep disorder *N* = 8225 (26%)	*p*
Age, *n* (%)				
≤ 60	22,159 (69%)	16,801 (71%)	5358 (65%)	< 0.001
> 60	9761 (31%)	6894 (29%)	2867 (35%)	
Sex, *n* (%)				
Male	15,500 (49%)	12,097 (51%)	3403 (41%)	< 0.001
Female	16,420 (51%)	11,598 (49%)	4822 (59%)	
Race, *n* (%)				
Non‐Hispanic White	14,076 (44%)	9, 715 (41%)	4361 (53%)	< 0.001
Non‐Hispanic Black	6808 (21%)	5118 (22%)	1690 (21%)	
Mexican American	4843 (15%)	3987 (17%)	856 (10%)	
Other	6193 (19%)	4875 (21%)	1318 (16%)	
Edu, *n* (%)				
Less than high school	7580 (24%)	5791 (24%)	1789 (22%)	< 0.001
High school grad or equivalent	7378 (23%)	5430 (23%)	1948 (24%)	
Some college or above	16,962 (53%)	12,474 (53%)	4488 (55%)	
Marriage, *n* (%)				
Married	16,521 (52%)	12,588 (53%)	3933 (48%)	< 0.001
Other	15,399 (48%)	11,107 (47%)	4292 (52%)	
PIR	2.52 ± 1.62	2.54 ± 1.62	2.48 ± 1.64	0.004
BMI, kg/m^2^	29.25 ± 7.01	28.78 ± 6.63	30.62 ± 7.84	< 0.001
Activity, *n* (%)	21,242 (67%)	15,915 (67%)	5327 (65%)	< 0.001
Smoking, *n* (%)				
Never	17,510 (55%)	13,733 (58%)	3777 (46%)	< 0.001
Former	7834 (25%)	5463 (23%)	2371 (29%)	
Current	6, 576 (21%)	4499 (19%)	2077 (25%)	
Diabetes, *n* (%)	4029 (13%)	2507 (11%)	1522 (19%)	< 0.001
Hypertension, *n* (%)	11,362 (36%)	7290 (31%)	4072 (50%)	< 0.001
ω‐3, g	1.76 ± 1.13	1.78 ± 1.14	1.71 ± 1.11	< 0.001
ω‐6, g	15.78 ± 9.26	15.89 ± 9.28	15.48 ± 9.18	< 0.001
(ω‐3) + (ω‐6), g	17.55 ± 10.19	17.67 ± 10.22	17.19 ± 10.10	< 0.001
(ω‐3)/(ω‐6), g	0.12 ± 0.06	0.12 ± 0.06	0.12 ± 0.05	0.040

*Note:* ω‐3: Total omega‐3 FA intake; ω‐6: Total omega‐6 FA intake; (ω‐3) + (ω‐6): the sum of omega‐3 FA intake and omega‐6 FA intake; (ω‐3)/(ω‐6): the ratio of omega‐3 FA intake to omega‐6 FA intake.

Abbreviations: BMI, body mass index; PIR, ratio of family income to poverty.

The intake of ω‐3 FA, ω‐6 FA, and the sum of ω‐3 FA and ω‐6 FA was significantly lower in the sleep disorder group than in the no sleep disorder group (*p* < 0.001). However, the ratio of ω‐3 FA intake to ω‐6 FA intake had a *p*‐value of 0.04 comparing the Sleep disorder group with the No sleep disorder group, which is close to the critical value.

Participants in the sleep disorder group were more likely to be female, aged ≤ 60 years, white, college educated, unmarried, have lower PIR, higher BMI, moderate or vigorous activity, never smoked, and not have diabetes.

Participants were stratified into tertiles based on ω‐3 FA intake levels: T1 (ω‐3 ≤ 1.17 g), T2 (1.17 g < ω‐3 ≤ 1.95 g), and T3 (ω‐3 > 1.95 g). Specifically, the ω‐3 FA intake data was rank‐ordered in ascending sequence, with the 33rd and 66th percentiles serving as demarcation thresholds for categorization. The baseline characteristics of the variables between groups were shown in Table 3. Using identical methodology, participants were stratified into tertiles based on ω‐6 FA intake: T1 (ω‐6 ≤ 10.86 g), T2 (10.86 g < ω‐6 ≤ 11.75 g), and T3 (ω‐6 > 11.72 g). The baseline characteristics of the variables between groups were shown in Table 4.

**TABLE 3:** Baseline characteristics by categories of omega (ω‐3) FA intake.

Characteristic	Overall *N* = 31,920	T1 (ω‐3 ≤ 1.17 g) *N* = 10,538 (33%)	T2 (1.17 g < ω‐3 ≤ 1.95 g) *N* = 10,853 (34%)	T3 (ω‐3 > 1.95 g) *N* = 10,529 (33%)	*p*
Age, *n* (%)					
≤ 60	22,159 (69%)	6866 (65%)	7557 (70%)	7736 (73%)	< 0.001
> 60	9761 (31%)	3672 (35%)	3296 (30%)	2793 (27%)	
Sex, *n* (%)					
Male	15,500 (49%)	4043 (38%)	5210 (48%)	6247 (59%)	< 0.001
Female	16,420 (51%)	6495 (62%)	5643 (52%)	4282 (41%)	
Race, *n* (%)					
Non‐Hispanic White	14,076 (44%)	4485 (43%)	4911 (45%)	4680 (44%)	< 0.001
Non‐Hispanic Black	6808 (21%)	2, 275 (22%)	2209 (20%)	2324 (22%)	
Mexican American	4843 (15%)	1757 (17%)	1, 626 (15%)	1460 (14%)	
Other	6193 (19%)	2021 (19%)	2107 (19%)	2065 (20%)	
Edu, *n* (%)					
Less than high school	7580 (24%)	3194 (30%)	2479 (23%)	1907 (18%)	< 0.001
High school grad or equivalent	7378 (23%)	2490 (24%)	2542 (23%)	2346 (22%)	
Some college or above	16,962 (53%)	4854 (46%)	5832 (54%)	6276 (60%)	
Marriage, *n* (%)					
Married	16,521 (52%)	5207 (49%)	5654 (52%)	5660 (54%)	< 0.001
Other	15,399 (48%)	5331 (51%)	5199 (48%)	4869 (46%)	
PIR	2.52 ± 1.62	2.27 ± 1.55	2.57 ± 1.63	2.73 ± 1.65	< 0.001
BMI, kg/m^2^	29.25 ± 7.01	29.31 ± 7.01	29.13 ± 6.84	29.32 ± 7.17	0.3
Activity, *n* (%)	21,242 (67%)	6357 (60%)	7313 (67%)	7572 (72%)	< 0.001
Smoking, *n* (%)					
Never	17,510 (55%)	5777 (55%)	6068 (56%)	5665 (54%)	< 0.001
Former	7834 (25%)	2429 (23%)	2644 (24%)	2761 (26%)	
Current	6, 576 (21%)	2332 (22%)	2141 (20%)	2103 (20%)	
Diabetes, *n* (%)	4029 (13%)	1491 (14%)	1303 (12%)	1235 (12%)	< 0.001
Hypertension, *n* (%)	11,362 (36%)	4066 (39%)	3764 (35%)	3532 (34%)	< 0.001
Sleep disorder, *n* (%)	8225 (26%)	2935 (28%)	2695 (25%)	2595 (25%)	< 0.001

Abbreviations: BMI, body mass index; PIR, ratio of family income to poverty.

**TABLE 4:** Baseline characteristics by categories of omega‐6 (ω‐6) FA intake.

Characteristic	Overall *N* = 31,920	T1 (ω‐6 ≤ 10.86 g) *N* = 10,536 (33%)	T2 (10.86 g < ω‐6 ≤ 11.75 g) *N* = 10,851 (34%)	T3 (ω‐6 > 11.72 g) *N* = 10,533 (33%)	*p*
Age, *n* (%)					
≤ 60	22,159 (69%)	6598 (63%)	7540 (69%)	8021 (76%)	< 0.001
> 60	9761 (31%)	3938 (37%)	3311 (31%)	2, 512 (24%)	
Sex, *n* (%)					
Male	15,500 (49%)	3931 (37%)	5118 (47%)	6451 (61%)	< 0.001
Female	16,420 (51%)	6605 (63%)	5733 (53%)	4082 (39%)	
Race, *n* (%)					
Non‐Hispanic White	14,076 (44%)	4341 (41%)	4918 (45%)	4817 (46%)	< 0.001
Non‐Hispanic Black	6808 (21%)	2117 (20%)	2189 (20%)	2502 (24%)	
Mexican American	4843 (15%)	1698 (16%)	1680 (15%)	1465 (14%)	
Other	6193 (19%)	2380 (23%)	2064 (19%)	1749 (17%)	
Edu, *n* (%)					
Less than high school	7580 (24%)	3277 (31%)	2449 (23%)	1854 (18%)	< 0.001
High school grad or equivalent	7378 (23%)	2463 (23%)	2522 (23%)	2, 393 (23%)	
Some college or above	16,962 (53%)	4796 (46%)	5880 (54%)	6286 (60%)	
Marriage, *n* (%)					
Married	16,521 (52%)	5238 (50%)	5806 (54%)	5477 (52%)	< 0.001
Other	15,399 (48%)	5298 (50%)	5045 (46%)	5056 (48%)	
PIR	2.52 ± 1.62	2.27 ± 1.57	2.58 ± 1.62	2.72 ± 1.65	< 0.001
BMI, kg/m^2^	29.25 ± 7.01	29.19 ± 6.85	29.06 ± 6.84	29.52 ± 7.31	0.001
Activity, *n* (%)	21,242 (67%)	6314 (60%)	7332 (68%)	7596 (72%)	< 0.001
Smoking, *n* (%)					
Never	17,510 (55%)	5924 (56%)	6046 (56%)	5540 (53%)	< 0.001
Former	7834 (25%)	2438 (23%)	2655 (24%)	2741 (26%)	
Current	6, 576 (21%)	2174 (21%)	2150 (20%)	2252 (21%)	
Diabetes, *n* (%)	4029 (13%)	1521 (14%)	1318 (12%)	1190 (11%)	< 0.001
Hypertension, *n* (%)	11,362 (36%)	4164 (40%)	3746 (35%)	3452 (33%)	< 0.001
Sleep disorder, *n* (%)	8225 (26%)	2865 (27%)	2726 (25%)	2634 (25%)	< 0.001

Abbreviations: BMI, body mass index; PIR, ratio of family income to poverty.

Participants with higher ω‐3 FA intake and ω‐6 FA intake in the population included in the study were more likely to have the following characteristics: male, age ≤ 60 years, white, college education or higher, married, higher PIR values, moderate or vigorous activity, never smoked, and no diabetes. In addition, the proportion of participants with sleep disorders was significantly lower in the T2 and T3 groups than in the T1 group for ω‐3 FA intake and ω‐6 FA intake (*p* < 0.001).

### 3.2 Dose–Response Relationship Analysis

**FIGURE 2:**
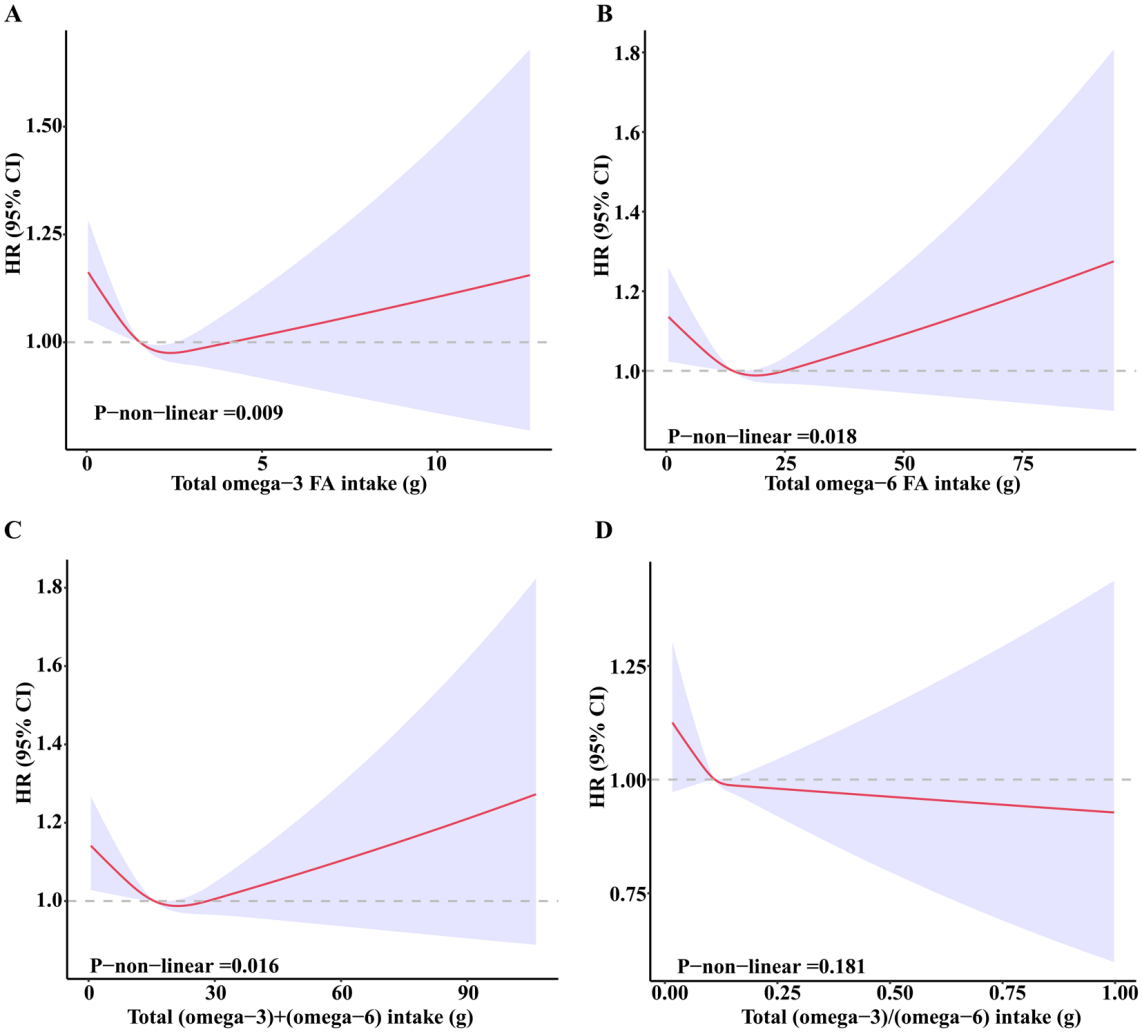
Restricted cubic spline model of FA intake and sleep disorders. (A) Restricted cubic spline plot of ω‐3 FA intake and sleep disorders. (B) Restricted cubic spline plot of ω‐6 FA intake and sleep disorders. (C) Restricted cubic spline plot of (ω‐3) + (ω‐6) FA intake and sleep disorders. (D) Restricted cubic spline plot of (ω‐3)/(ω‐6) FA intake and sleep disorders.

Dose–response relationship analysis was performed using restricted cubic spline, and the results were shown in Figure 2. ω‐3 FA intake, ω‐6 FA intake, the sum of ω‐3 FA intake, and ω‐6 FA intake were related to sleep disorders in a U‐shape with significant nonlinear characteristics (Pnonlinear < 0.05); whereas the relationship between the ratio of ω‐3 FA intake to ω‐6 FA intake and sleep disorders was L‐shaped (Pnonlinear = 0.181).

### 3.3 Logistics Regression Analysis

Three logistic regression analysis models were developed separately to assess the four variables of ω‐3 FA intake, ω‐6 FA intake, the sum of ω‐3 and ω‐6 FA intake, the ratio of ω‐3 to ω‐6 FA intake, and sleep disorders. The results were shown in Table 5.

**TABLE 5:** Multivariate Cox regression analysis of FA intake and sleep disorders.

Quintiles	Model 1			Model 2			Model 3		
	HR	95% CI	*p*	HR	95% CI	*p*	HR	95% CI	*p*
Total ω‐3 FA intake (g)			< 0.001			< 0.001			0.005
T1 (ω‐3 ≤ 1.17 g)	—	—		—	—		—	—	
T2 (1.17 g < ω‐3 ≤ 1.95 g)	0.86	0.81, 0.91		0.89	0.83, 0.94		0.91	0.85, 0.96	
T3 (ω‐3 > 1.95 g)	0.85	0.80, 0.90		0.92	0.86, 0.98		0.93	0.87, 0.99	
P for trend			< 0.001			0.007			0.026
Total ω‐6 FA intake (g)			< 0.001			0.3			0.3
T1 (ω‐6 ≤ 10.86 g)	—	—		—	—		—	—	
T2 (10.86 g < ω‐6 ≤ 11.75 g)	0.90	0.84, 0.96		0.95	0.89, 1.02		0.95	0.89, 1.02	
T3 (ω‐6 > 11.72 g)	0.89	0.84, 0.95		0.98	0.92, 1.04		0.98	0.92, 1.04	
P for trend			< 0.001			0.247			0.468
(ω‐3) + (ω‐6) FA intake (g)			< 0.001			0.092			0.3
T1 (≤ 12.16 g)	—	—		—	—		—	—	
T2 (12.16 g ~ 19.89 g)	0.90	0.85, 0.96		0.93	0.88, 1.00		0.95	0.89, 1.02	
T3 (> 19.89 g)	0.88	0.83, 0.93		0.95	0.89, 1.01		0.96	0.90, 1.02	
P for trend			< 0.001			0.083			0.205
(ω‐3)/(ω‐6) FA intake (g)			0.057			0.2			0.2
T1 (≤ 0.099)	—	—		—	—		—		
T2 (0.099 ~ 0.123)	1.01	0.95, 1.08		0.99	0.93, 1.06		1.00	0.93, 1.07	
T3 (> 0.123)	0.94	0.89, 1.00		0.95	0.89, 1.00		0.95	0.89, 1.01	
P for trend			0.046			0.063			0.077

*Note:* Model 1 was adjusted for none; Model 2 was adjusted for Age, Sex, Race, Edu, Marriage, PIR, BIM; Model 3 was adjusted for Age, Sex, Race, Edu, Marriage, PIR, BIM, Activity, Smoking, Diabetes; Hypertension.

Abbreviations: CI, confidence interval; HR, hazard ratio.

In the unadjusted model 1, the three variables ω‐3 FA intake, ω‐6 FA intake, and the sum of ω‐3 and ω‐6 FA intake were significantly associated with sleep disorders (*p* < 0.001, *p* for trend < 0.001), whereas the ratio of ω‐3 to ω‐6 FA intake was not similarly associated with sleep disorders (*p* = 0.057, *p* for trend = 0.046).

In model 3, only ω‐3 FA intake was significantly associated with sleep disorders (*p* = 0.005, *p* for trend = 0.026). Using the T1 group (ω‐3 ≤ 1.17 g) as a reference, the OR (95% CI) for the T2 group (1.17 g < ω‐3 ≤ 1.95 g) was 0.91 (0.85–0.96), indicating that participants in the T2 group had a 9% reduced risk of having sleep disorders compared with the T1 group. The OR (95% CI) for the T3 group (ω‐3 > 1.95 g) was 0.93 (0.87–0.99), indicating that participants in the T3 group had a 7% reduced risk of suffering from sleep disorders compared to the T1 group. However, the association between ω‐6 FA intake, the sum of ω‐3 and ω‐6 FA intake, and the ratio of ω‐3 to ω‐6 FA intake and sleep disorders was not significant (*p* > 0.05, *p* for trend > 0.05).

In addition, the calculation results of model 2 were basically consistent with those of model 3.

### 3.4 Subgroup Analyses

**FIGURE 3:**
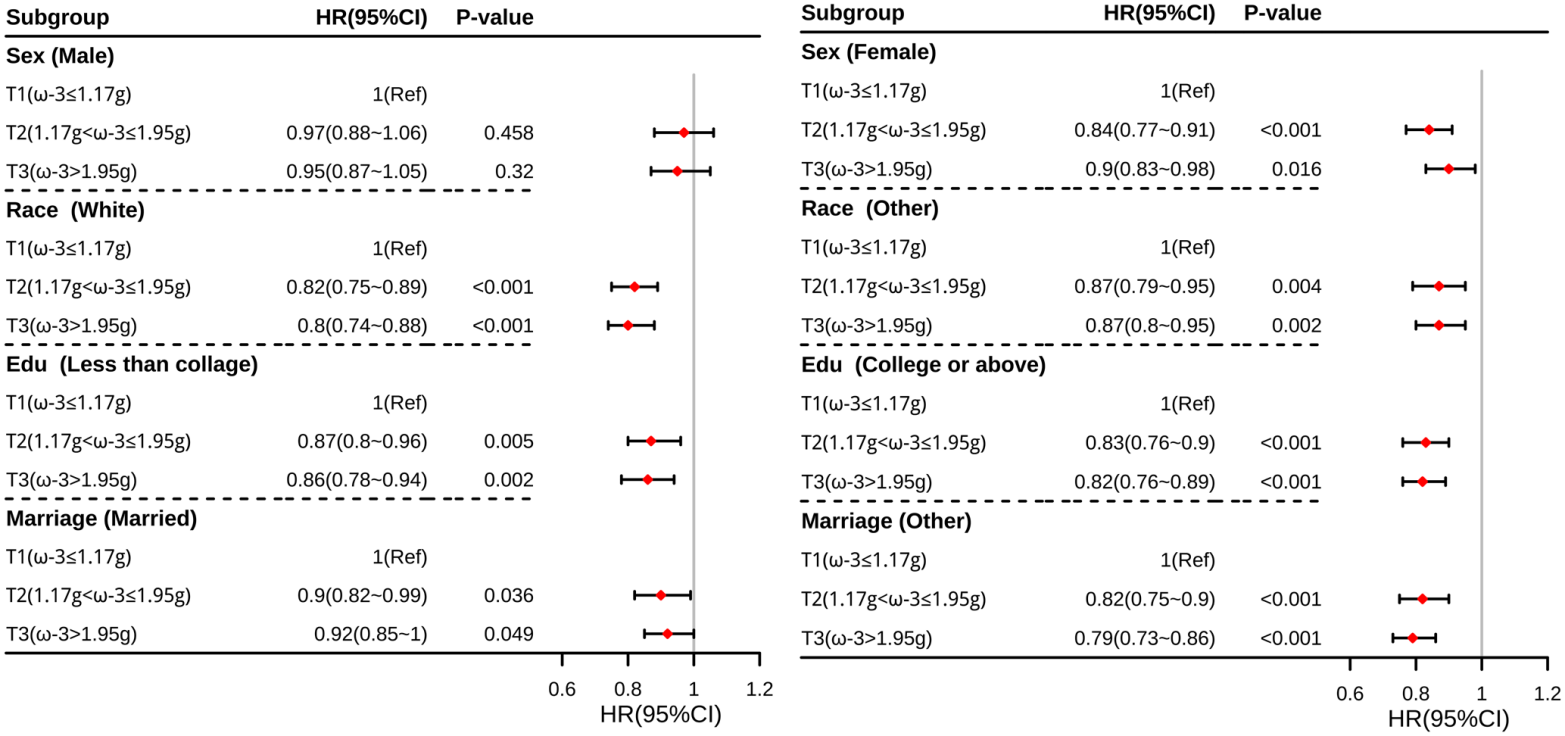
Forest plots of subgroup analyses.

Subgroup analyses were used to assess whether the association between ω‐3 FA intake and sleep disorders differed between subgroups. Subgroup stratification criteria were used for sex (male or female), race (non‐Hispanic white or other), education (less than college or higher), and marriage (married or other). The results of the subgroup analyses were shown in Figure 3. The association between ω‐3 FA intake and sleep disorders was not significant in the male subgroup (*p* > 0.05), while it was significant in all other subgroups (*p* < 0.05), and the results of the analyses were basically the same, showing good stability.

### 3.5 Mendelian Randomization (MR) Estimates

**FIGURE 4:**
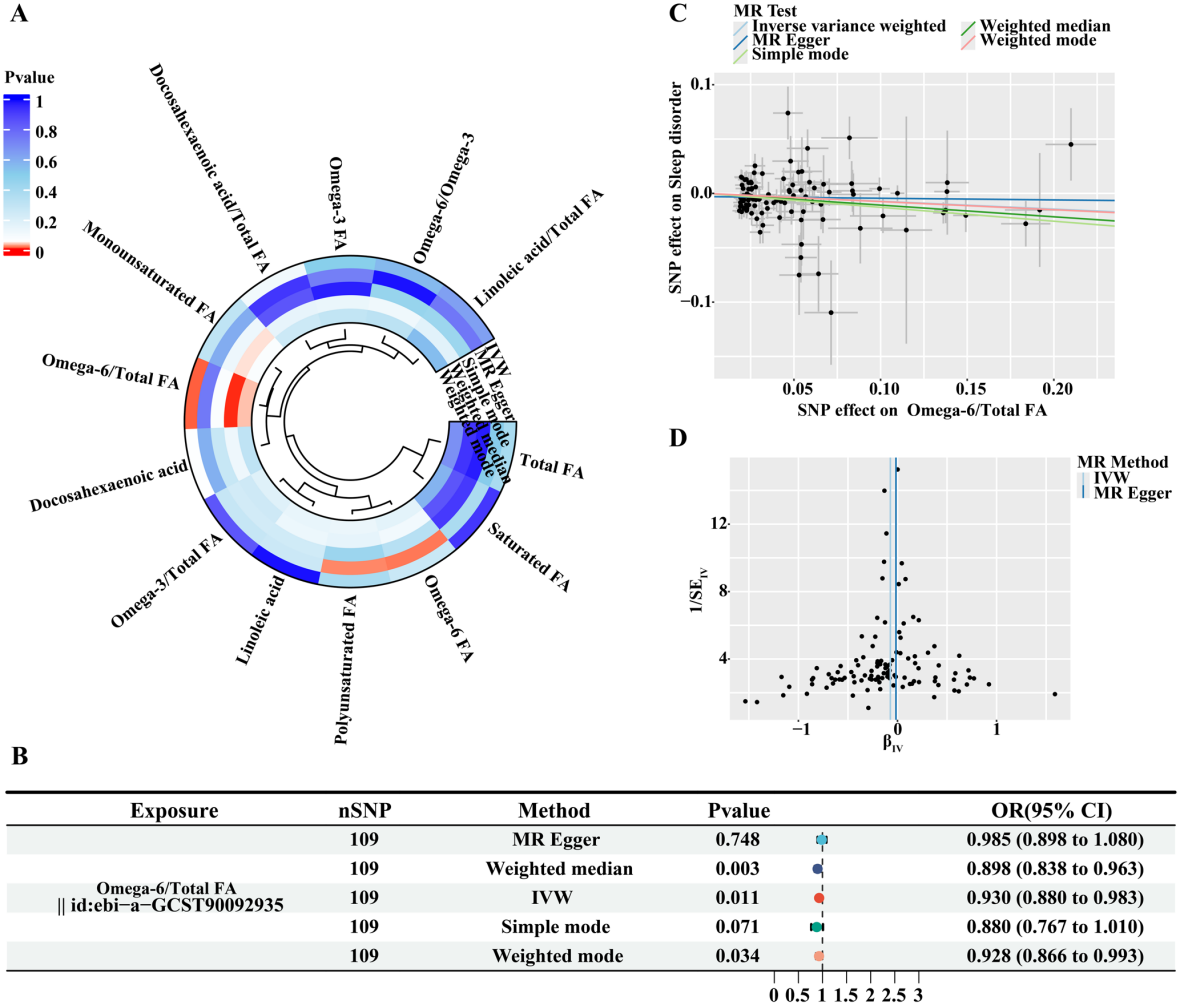
MR analysis showing a causal effect of omega‐6/total FA on sleep disorders. (A) Circos plot of MR analysis results for omega‐3 FA, omega‐6 FA, omega‐6/omega‐3, omega‐3/total FA, omega‐6/total FA, docosahexaenoic acid, docosahexaenoic acid/total FA, linoleic acid, linoleic acid/total FA, monounsaturated FA, polyunsaturated FA, saturated FA, and total FA. (B) Forest plot of causal effect estimates in MR analysis. SNP, single‐nucleotide polymorphism; IVW, inverse variance weighted. (C) Scatter plot of causal effect estimates for omega‐6/total FA on sleep disorders. (D) Funnel plot of causal effect estimates for omega‐6/total FA on sleep disorders.

Causal associations between 13 fatty‐acid‐related traits and risk of sleep disorders were analyzed using five MR methods including IVW, MR Egger, simple mode, weighted median, and weighted mode. P values of these results were shown in Figure 4A. Based on IVW results, among the 13 fatty‐acid‐related traits, omega‐6/total FA was found causally associated with risk of sleep disorders (IVW OR = 0.930, 95% CI: 0.880–0.983, *p* = 0.011; Figure 4B). The results of the weighted median and weighted mode also supported this causal relationship (weighted median OR = 0.898, 95% CI: 0.838–0.963, *p* = 0.003; weighted mode OR = 0.928, 95% CI: 0.866–0.993, *p* = 0.034; Figure 4B). The results were further displayed in a scatterplot in Figure 4C. The five different colored lines represent the five methods of MR analysis, with the slope of each line indicating the direction of the causal relationship (Figure 4C). According to the OR value and the scatterplot results, omega‐6/total FA was a protective factor for sleep disorders. The funnel plot showed that the distribution of scatter points was generally symmetrical, indicating that there was no significant bias in the results of the study (Figure 4D).

### 3.6 Sensitivity Analyses

Sensitivity analyses, including heterogeneity Cochran's *Q* tests using the MR‐Egger and IVW methods, and pleiotropy test using the Egger_intercept method, were performed and the results were shown in Table 6. For the exposure omega‐6/total FA, the *p* values of the MR‐Egger and IVW Cochran's *Q* tests were greater than 0.05 (MR‐Egger *Q* = 104.020, MR‐Egger *p* = 0.103; IVW *Q* = 105.547, IVW *p* = 0.098; Table 6), indicating that no heterogeneity was found. The *p* value of the Egger_intercept was also greater than 0.05, suggesting the absence of horizontal pleiotropy (Egger_intercept = 2.056E‐03, *p* = 0.262; Table 6). In addition, the leave‐one‐out analysis showed that the removal of any single SNP did not have an impact on the results (Figure S1).

**TABLE 6:** Heterogeneity and pleiotropy tests of the MR analysis.

Exposure	Heterogeneity test				Pleiotropy test	
	MREgger_Q	MREgger_P	IVW_Q	IVW_P	Egger _intercept	*p*
Omega‐3 FA	162.157	3.514E‐06	162.250	4.712E‐06	−3.922E‐04	0.822
Omega‐6 FA	85.906	0.422	90.825	0.313	3.904E‐03	0.031
Omega‐6/Omega‐3	127.702	1.068E‐04	128.476	1.203E‐04	1.199E‐03	0.505
Omega‐3/Total FA	100.155	4.925E‐04	102.955	3.483E‐04	−3.092E‐03	0.208
Omega‐6/Total FA	**104.020**	**0.103**	**105.547**	**0.098**	**2.056E‐03**	**0.262**
Docosahexaenoic acid	105.379	5.050E‐03	116.799	6.635E‐04	−5.034E‐03	0.007
Docosahexaenoic acid/Total FA	78.455	0.140	82.304	0.099	−3.289E‐03	0.077
Linoleic acid	84.574	0.313	86.569	0.288	2.608E‐03	0.176
Linoleic acid/Total FA	138.125	6.603E‐04	139.094	6.959E‐04	−1.510E‐03	0.431
Monounsaturated FA	189.054	7.513E‐05	189.077	9.492E‐05	1.987E‐04	0.904
Polyunsaturated FA	113.553	0.107	119.660	0.059	3.954E‐03	0.025
Saturated FA	183.286	6.289E‐06	187.126	3.582E‐06	−2.933E‐03	0.137
Total FA	130.019	0.043	132.583	0.036	2.388E‐03	0.155

*Note:* The bolded text represents the results of heterogeneity and pleiotropy tests for the causally‐associated Omega‐6/Total FA (exposure) and sleep disorder (outcome) identified through Mendelian randomization analysis in Figure 4.

## 4 Discussion

The current study, leveraging data from the National Health and Nutrition Examination Survey (NHANES) from 2005 to 2018, provided a comprehensive examination of the relationship between omega‐3 (ω‐3) and omega‐6 (ω‐6) polyunsaturated fatty acid intake and sleep disorders among US adults. Our findings revealed that participants with higher ω‐3 FA intake had a reduced risk of sleep disorders, aligning with a growing body of literature that suggested a beneficial role of ω‐3 FA in sleep health (Montgomery et al. 2014; Del Brutto et al. 2016). However, our Mendelian randomization (MR) analysis indicated that a higher ratio of ω‐6 to total fatty acid levels was causally associated with a lower risk of sleep disorders (IVW OR = 0.930, 95% CI: 0.880–0.983, *p* = 0.011), a novel finding that adds depth to our understanding of the role of dietary fats in sleep modulation (Murphy et al. 2022). The results yielded a significant contribution to the field, given the inconsistent results from observational studies (Reutrakul and Van Cauter 2018).

Sleep is an indispensable physiological phenomenon for humans and is influenced by a variety of environmental factors and life‐history traits (Allada and Siegel 2008; Joiner 2016). The relationship between polyunsaturated fatty acid (PUFA) intake and sleep has been the subject of extensive research, with findings that have been inconsistent. The ω‐3 FA family, consisting mainly of alpha‐linolenic acid, eicosapentaenoic acid (EPA) and docosahexaenoic acid (DHA), is known for its anti‐inflammatory and anti‐oxidative properties (Oh et al. 2010; Weldon et al. 2007; Xue et al. 2012). Several animal studies revealed that ω‐3 FA may be associated with sleep onset and maintenance by regulating melatonin composition and neuronal membrane structure (Catala 2010; Zhang et al. 1998; Kim et al. 1999; Yehuda et al. 1998). While some studies have reported a positive association between ω‐3 FA levels and human sleep quality (Montgomery et al. 2014; Scorza et al. 2013), others have found no association or even a negative association (Cohen et al. 2014; Hansen et al. 2014; Zhang et al. 2022). For instance, a cross‐sectional study employing bootstrap and rolling‐window analyses by Liu et al. demonstrated that DHA intake was associated with improved sleep duration among women aged > 44 years (Liu et al. 2024). The discrepancies between their findings and ours may stem from several key factors. First, the studies assessed distinct sleep‐related outcomes: while our investigation examined general sleep disorders, Liu et al. specifically focused on very short sleep duration (< 5 h). Second, the study populations differed substantially—our analysis encompassed a broad adult cohort, whereas Liu et al. conducted age‐ and sex‐stratified analyses, uncovering more nuanced associations. Third, methodological differences likely contributed to divergent results; notably, our Mendelian randomization approach contrasts with their rolling‐window and bootstrap techniques. Supporting evidence from randomized controlled trials (RCTs) suggested that DHA supplementation enhances sleep quality in healthy adults (Patan et al. 2021; Yokoi‐Shimizu et al. 2022; Jackson 2025). In contrast, interventions rich in EPA showed no significant effects in specific subgroups, such as menopausal women and migraine sufferers (Jackson 2025). Furthermore, Gangitano et al. synthesized evidence indicating that high‐fat diets—particularly those rich in saturated fats—may adversely affect sleep quality by inducing circadian desynchrony and disrupting metabolic and clock‐related gene expression (Gangitano et al. 2023). However, the effects of unsaturated fatty acids (e.g., ω‐3 and ω‐6 PUFAs) on sleep architecture remain inconclusive (Gangitano et al. 2023). The findings of our study align more closely with those of the former group, indicating a beneficial role of ω‐3 FA in sleep health. Overall, the discrepancy in these findings may be attributed to variations in study design, population characteristics, and the methods used to assess sleep disorders. Our use of Mendelian randomization (MR) analysis to evaluate causal effects represents a significant methodological advancement over simpler observational studies (Burgess et al. 2012). A recent MR study by Zuo et al. explored the causal relationships among insomnia, circulating fatty acids, and heart failure (Zuo et al. 2025). Their findings revealed that genetically predicted insomnia significantly elevated the risk of heart failure, with fatty acids—particularly saturated and monounsaturated types—mediating this association. Interestingly, while higher ω‐3 levels were inversely correlated with insomnia, they exhibited a positive association with heart failure risk (Zuo et al. 2025). These results contrast with our MR analysis, which identified a protective effect of a higher ω‐6 to total fatty acid ratio against sleep disorders. The discrepancies between the two studies may stem from several factors. Firstly, the outcome measure in their study was insomnia, while our study focused on a broader concept of sleep disorders, which may include various types of sleep‐related problems. Different definitions and measurements of the outcome can lead to different results. Secondly, unmeasured confounders, such as mental health status in sleep research or metabolic dysfunction in cardiovascular studies, could differentially bias the observed associations. Thirdly, the scope of fatty acids examined varies: Zuo et al. assessed a broad spectrum of fatty acids within a complex cardiometabolic pathway, whereas our study specifically investigated polyunsaturated fatty acids (PUFAs) and their ratios in relation to sleep health.

Nevertheless, the function of ω‐6 FA in sleep remains uncertain. Some studies have proposed that ω‐6 FA may have a distinct role in sleep modulation compared to ω‐3 FA (Tang et al. 2018). Our findings contrast with those of some previous studies, which have suggested a positive association between ω‐6 intake/levels and sleep disorders (Luo et al. 2021; Sanders et al. 2023). This discrepancy may be attributable to differences in dietary patterns, genetic factors, or the specific types of ω‐6 FA consumed, and further investigation is warranted.

One of the key strengths of our study was the large, nationally representative sample of US adults, which allowed for greater generalizability of our findings. Furthermore, the utilization of sophisticated statistical techniques, including multivariate regression and MR analysis, facilitated a more intricate comprehension of the interrelationship between PUFA consumption and sleep disorders, while accounting for potential confounding factors such as age, sex, race, and lifestyle variables (Hysing et al. 2018; Cornu et al. 2010). The application of restricted cubic spline analysis permitted the exploration of non‐linear relationships, which revealed a U‐shaped association between ω‐3 and ω‐6 FA intake and sleep disorders. This finding has not been widely reported in the literature (Luo et al. 2021). Another strength was the rigorous control for confounding variables, which enhanced the reliability of the results. A wide range of covariates was adjusted for, including socioeconomic status, physical activity, and smoking status, which have been known to influence both PUFA intake and sleep disorders (Reutrakul and Van Cauter 2018; Yaffe et al. 2014). The utilization of MR analysis served to reinforce the findings of this study, as it provided a methodology through which causality can be inferred from observational data. This represented a significant advancement over the methodology employed in traditional cross‐sectional studies (Burgess et al. 2012).

While the study has several strengths, it is important to acknowledge the limitations of the research. The cross‐sectional nature of the NHANES data precludes the establishment of causality and the inference of temporality in the relationship between PUFA intake and sleep disorders. Furthermore, dietary intake was evaluated through self‐reported 24‐h dietary recalls, which may be susceptible to recall bias and may not accurately reflect long‐term dietary habits (Zhong et al. 2024). Additionally, the generalizability of our findings may be limited by the fact that our study population is restricted to non‐institutionalized US civilians. Furthermore, the accuracy of the data is affected by recall bias, which represents a common issue in nutritional epidemiology. This is an inherent limitation of studies that rely on self‐reported dietary intake data, and our study is no exception. The use of objective measures of dietary intake, such as biomarkers, in future studies could help to address this limitation (Li et al. 2024). A further limitation is the potential for unmeasured confounding. Despite the adjustment for a comprehensive range of potential confounding variables, it is possible that other factors influencing both PUFA intake and sleep disorders were not fully accounted for in the analysis. For example, mental health conditions, such as depression and anxiety, have been demonstrated to impact both sleep and dietary habits. However, these factors were not fully captured in our study (Irmisch et al. 2007). In addition, it should be noted that the outcome measure of sleep disorders was based on self‐reported data, which may not be as accurate as clinical diagnoses. Future studies employing objective measures of sleep, such as polysomnography, may yield more precise estimates of the relationship between PUFA intake and sleep disorders (Xu et al. 2022).

The findings from our study have several implications for future research and public health practice. The results of this study indicated that an increase in ω‐3 FA intake and maintenance of a balanced ratio of ω‐6 to total fatty acids may be beneficial for sleep health. These findings have the potential to inform public health guidelines and contribute to the development of targeted nutritional interventions aimed at improving sleep quality and overall health (Jaqua et al. 2023). It would be beneficial for future research to aim to replicate these results in longitudinal studies and clinical trials, and to explore the mechanisms underlying the observed associations. Moreover, further interventional studies are required in order to establish optimal dietary recommendations for the prevention and treatment of sleep disorders. Moreover, future studies should endeavor to address the limitations of the present study by employing objective measures of dietary intake and sleep outcomes, and by accounting for potential confounding factors such as mental health conditions (Miller and Howarth 2023).

In conclusion, our study provides novel insights into the relationship between PUFA intake and sleep disorders, indicating the potential benefits of dietary modification for sleep health. Although our findings are encouraging, further research is required to ascertain causality and to develop evidence‐based dietary recommendations for the prevention and treatment of sleep disorders.

## Author Contributions

**Lin Wang:** writing – original draft, data curation, methodology, project administration, software, funding acquisition. **Wei Quan:** writing – original draft, data curation, methodology, project administration, software. **Jia Song, Yidan Qin, Huibin Zeng, Jian Zhang, Xuan Zhao:** data curation, investigation, formal analysis. **Jia Li:** supervision, validation, writing – review and editing. **Jiajun Chen:** conceptualization, supervision, validation, writing and review and editing, funding acquisition.

## Ethics Statement

Ethical review and approval were not required for this study because the data used in this study was public, anonymized, and de‐identified.

## Conflicts of Interest

The authors declare no conflicts of interest.

## Supporting information


**Figure S1.** The MR leave‐one‐out analysis for “Omega‐6/Total FA” on “Sleep disorders.”

**Table S1.** STROBE‐MR checklist.

## Data Availability

The data that support the findings of this study are openly available in NHANSE at https://www.cdc.gov/nchs/nhanes/ and MRCIEU GWAS at https://gwas.mrcieu.ac.uk/.
