# Supplementary figures and images for: Association Between ω‐3, ω‐6 Polyunsaturated Fatty Acid and Sleep Disorders: From Cross‐Sectional to Mendelian Randomization Studies
Source: Food Sci Nutr. 2025 May 27;13(6):e70311. doi: 10.1002/fsn3.70311 (PMC12108440; doi:10.1002/fsn3.70311)

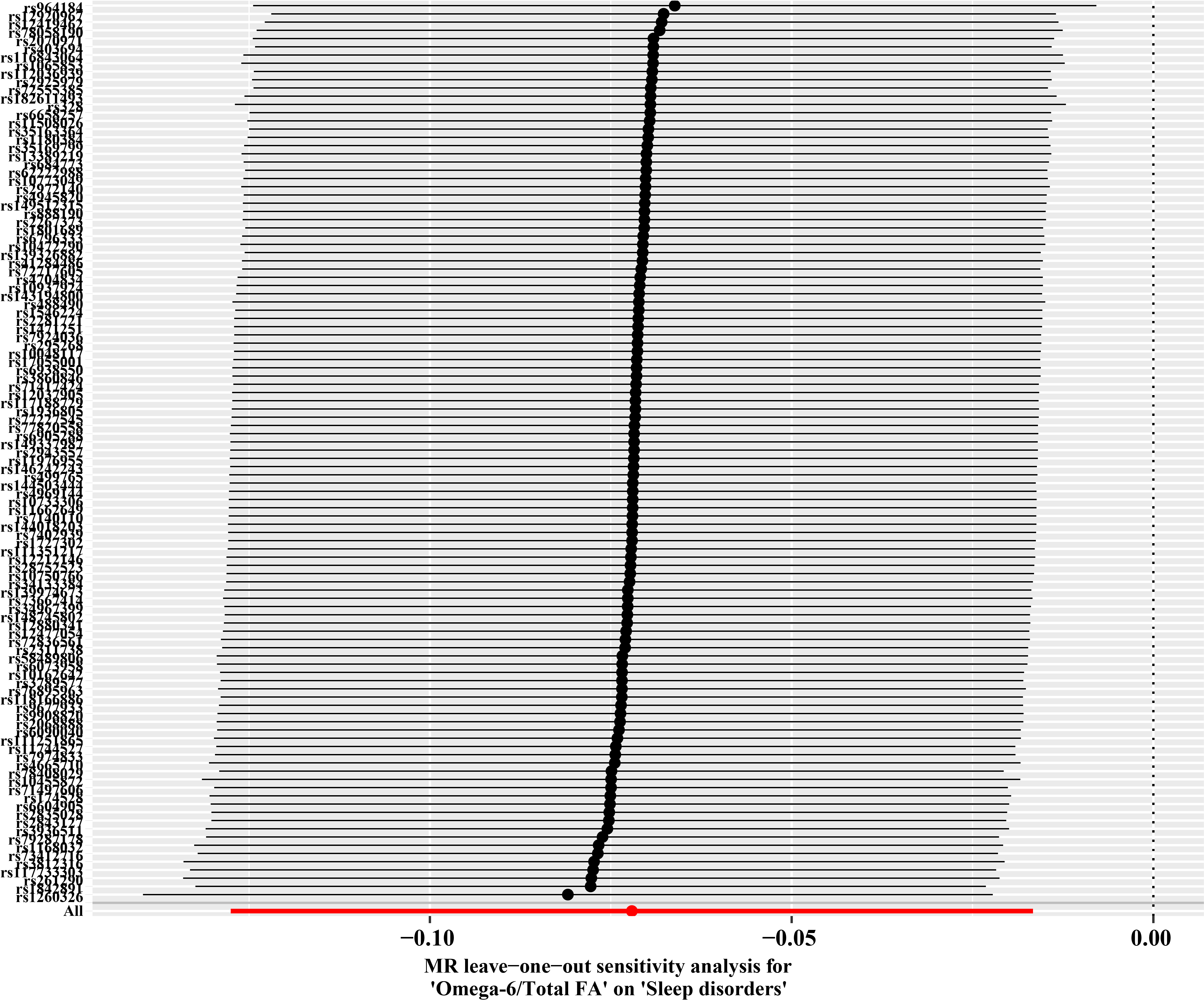

Supplement: Supplementary file 1 — Figure S1. The MR leave‐one‐out analysis for “Omega‐6/Total FA” on “Sleep disorders.” [file FSN3-13-e70311-s001.png]
