# Supplementary material for: Association Between ω‐3, ω‐6 Polyunsaturated Fatty Acid and Sleep Disorders: From Cross‐Sectional to Mendelian Randomization Studies
Source: Food Sci Nutr. 2025 May 27;13(6):e70311. doi: 10.1002/fsn3.70311 (PMC12108440; doi:10.1002/fsn3.70311)
Supplement: Supplementary file 2 — Table S1. STROBE‐MR checklist. [file FSN3-13-e70311-s002.docx]

**STROBE-MR checklist of recommended items to address in reports of Mendelian randomization studies**^1^ ^2^

| **Item No.** | **Section** | **Checklist item** | **Page No.** | **Relevant text from manuscript** |
| --- | --- | --- | --- | --- |
| 1 | **TITLE and ABSTRACT** | Indicate Mendelian randomization (MR) as the study’s design in the title and/or the abstract if that is a main purpose of the study | 1 | Association between ω-3, ω-6 polyunsaturated fatty acid and sleep disorders: From cross-sectional to Mendelian randomization studies |
|  | **INTRODUCTION** |  |  |  |
| 2 | **Background** | Explain the scientific background and rationale for the reported study. What is the exposure? Is a potential causal relationship between exposure and outcome plausible? Justify why MR is a helpful method to address the study question | 2 | Sleep disorders are increasingly being acknowledged as a significant public health concern, affecting millions of individuals across the globe. In the United States alone, it is estimated that approximately 50 to 70 million adults experience some form of sleep disorder [1]. These disorders are associated with various adverse health outcomes, including cardiovascular diseases, diabetes, and impaired cognitive function [2-4]. Despite the availability of diagnostic tools and treatments such as pharmacological treatments and behavioral interventions, many sleep disorders remain underdiagnosed and undertreated [5, 6]. This highlights the necessity for further research into the factors influencing sleep health and the development of more effective intervention strategies.  Polyunsaturated fatty acids (PUFAs), particularly omega-3 (ω-3) and omega-6 (ω-6) fatty acids, have been the subject of considerable interest with regard to their potential role in the modulation of sleep health. ω-3 PUFAs, which are commonly found in fish oil, have anti-inflammatory properties and are essential for brain health [7]. On the other hand, ω-6 PUFAs, which are found in vegetable oils, have been identified as precursors to pro-inflammatory molecules [8]. It is thought that the balance between these fatty acids exerts an influence on a number of physiological processes, including sleep. However, the relationship between ω-3 and ω-6 and sleep disorders are controversial. A series of researches indicated that the ω-3 FA level was positively associated with sleep quality [9-13]. |
| 3 | **Objectives** | State specific objectives clearly, including pre-specified causal hypotheses (if any). State that MR is a method that, under specific assumptions, intends to estimate causal effects | 3 | Then a two-sample MR analysis was applied to evaluate whether there were causal effects of multiple circulating fatty acids (omega-3 FA, omega-6 FA, omega-6/omega-3, omega-3/total FA, omega-6/total FA, docosahexaenoic acid, docosahexaenoic acid/total FA, linoleic acid, linoleic acid/total FA, monounsaturated FA, polyunsaturated FA, saturated FA, and total FA) on the risk of sleep disorders. |
|  | **METHODS** |  |  |  |
| 4 | **Study design and data sources** | Present key elements of the study design early in the article. Consider including a table listing sources of data for all phases of the study. For each data source contributing to the analysis, describe the following: | 4 | In this study, data from NHANSE (2005-2018) was used to obtain 70,190 participants. Then a two-sample MR analysis was performed to determine the causal impact of multiple circulating fatty acids on the risk of sleep disorders Figure 1B. |
|  | a) | Setting: Describe the study design and the underlying population, if possible. Describe the setting, locations, and relevant dates, including periods of recruitment, exposure, follow-up, and data collection, when available. | 4 | In this study, data from NHANSE (2005-2018) was used to obtain 70,190 participants. The outcome dataset of sleep disorders was obtained from the FinnGenR10 with 49,880 cases and 358,194 controls. |
|  | b) | Participants: Give the eligibility criteria, and the sources and methods of selection of participants. Report the sample size, and whether any power or sample size calculations were carried out prior to the main analysis | 4 | In this study, data from NHANSE (2005-2018) was used to obtain 70,190 participants. Firstly, 30,441 participants under the age of 20 were excluded. Secondly, 28 participants without sleep disorders were excluded. Then 4,423 participants without PUFA data were excluded. Finally, 3,378 participants with incomplete covariates were removed, leaving 31,920 participants included in the study.  The outcome dataset of sleep disorders was obtained from the FinnGenR10 with 49,880 cases and 358,194 controls. |
|  | c) | Describe measurement, quality control and selection of genetic variants | 4 | The selection of instrumental variables (IVs) was guided by the following 3 basic assumptions: (1) the selected IVs must be significantly associated with the exposure (P < 5E-06); (2) the IVs are independent from confounders that may influence the exposure and outcome; (3) the IVs can only influence the outcome through their effects on the exposure and cannot influence the outcome by other means (LD: r2 < 0.001within 10,000kb). |
|  | d) | For each exposure, outcome, and other relevant variables, describe methods of assessment and diagnostic criteria for diseases | 4 | Firstly, 30,441 participants under the age of 20 were excluded. Secondly, 28 participants without sleep disorders were excluded. Then 4,423 participants without PUFA data were excluded. Finally, 3,378 participants with incomplete covariates were removed, leaving 31,920 participants included in the study.  The selection of instrumental variables (IVs) was guided by the following 3 basic assumptions: (1) the selected IVs must be significantly associated with the exposure (P < 5E-06); (2) the IVs are independent from confounders that may influence the exposure and outcome; (3) the IVs can only influence the outcome through their effects on the exposure and cannot influence the outcome by other means (LD: r2 < 0.001within 10,000kb). |
|  | e) | Provide details of ethics committee approval and participant informed consent, if relevant | NA | NA |
| 5 | **Assumptions** | Explicitly state the three core IV assumptions for the main analysis (relevance, independence and exclusion restriction) as well assumptions for any additional or sensitivity analysis | 4 | The selection of instrumental variables (IVs) was guided by the following 3 basic assumptions: (1) the selected IVs must be significantly associated with the exposure (P < 5E-06); (2) the IVs are independent from confounders that may influence the exposure and outcome; (3) the IVs can only influence the outcome through their effects on the exposure and cannot influence the outcome by other means (LD: r2 < 0.001within 10,000kb). |
| 6 | **Statistical methods: main analysis** | Describe statistical methods and statistics used |  |  |
|  | a) | Describe how quantitative variables were handled in the analyses (i.e., scale, units, model) | 4 | Then a two-sample MR analysis was performed to determine the causal impact of multiple circulating fatty acids on the risk of sleep disorders Figure 1B. The selection of instrumental variables (IVs) was guided by the following 3 basic assumptions: (1) the selected IVs must be significantly associated with the exposure (P < 5E-06); (2) the IVs are independent from confounders that may influence the exposure and outcome; (3) the IVs can only influence the outcome through their effects on the exposure and cannot influence the outcome by other means (LD: r2 < 0.001within 10,000kb).  The GWAS data used for MR analysis were obtained from publicly available GWAS databases, and detailed information was provided in Table 1. The datasets for fatty-acid-related traits, including omega-3 FA, omega-6 FA, omega-6/omega-3, omega-3/total FA, omega-6/total FA, docosahexaenoic acid, docosahexaenoic acid/total FA, linoleic acid, linoleic acid/total FA, monounsaturated FA, polyunsaturated FA, saturated FA, and total FA, were obtained from the MRCIEU GWAS (https://gwas.mrcieu.ac.uk/) [21]. The outcome dataset of sleep disorders was obtained from the FinnGenR10 with 49,880 cases and 358,194 controls (https://storage.googleapis.com/finngen-public-data-r10/summary_stats/finngen_R10_SLEEP.gz) [22]. Missing data was deleted. The exposure and outcome data above are from European populations. |
|  | b) | Describe how genetic variants were handled in the analyses and, if applicable, how their weights were selected | 4 | Then a two-sample MR analysis was performed to determine the causal impact of multiple circulating fatty acids on the risk of sleep disorders Figure 1B. The selection of instrumental variables (IVs) was guided by the following 3 basic assumptions: (1) the selected IVs must be significantly associated with the exposure (P < 5E-06); (2) the IVs are independent from confounders that may influence the exposure and outcome; (3) the IVs can only influence the outcome through their effects on the exposure and cannot influence the outcome by other means (LD: r2 < 0.001within 10,000kb).  The GWAS data used for MR analysis were obtained from publicly available GWAS databases, and detailed information was provided in Table 1. The datasets for fatty-acid-related traits, including omega-3 FA, omega-6 FA, omega-6/omega-3, omega-3/total FA, omega-6/total FA, docosahexaenoic acid, docosahexaenoic acid/total FA, linoleic acid, linoleic acid/total FA, monounsaturated FA, polyunsaturated FA, saturated FA, and total FA, were obtained from the MRCIEU GWAS (https://gwas.mrcieu.ac.uk/) [22]. The outcome dataset of sleep disorders was obtained from the FinnGenR10 with 49,880 cases and 358,194 controls (https://storage.googleapis.com/finngen-public-data-r10/summary_stats/finngen_R10_SLEEP.gz) [23]. Missing data was deleted. The exposure and outcome data above are from European populations. |
|  | c) | Describe the MR estimator (e.g. two-stage least squares, Wald ratio) and related statistics. Detail the included covariates and, in case of two-sample MR, whether the same covariate set was used for adjustment in the two samples | 4 | Then a two-sample MR analysis was performed to determine the causal impact of multiple circulating fatty acids on the risk of sleep disorders Figure 1B. The selection of instrumental variables (IVs) was guided by the following 3 basic assumptions: (1) the selected IVs must be significantly associated with the exposure (P < 5E-06); (2) the IVs are independent from confounders that may influence the exposure and outcome; (3) the IVs can only influence the outcome through their effects on the exposure and cannot influence the outcome by other means (LD: r2 < 0.001within 10,000kb).  The GWAS data used for MR analysis were obtained from publicly available GWAS databases, and detailed information was provided in Table 1. The datasets for fatty-acid-related traits, including omega-3 FA, omega-6 FA, omega-6/omega-3, omega-3/total FA, omega-6/total FA, docosahexaenoic acid, docosahexaenoic acid/total FA, linoleic acid, linoleic acid/total FA, monounsaturated FA, polyunsaturated FA, saturated FA, and total FA, were obtained from the MRCIEU GWAS (https://gwas.mrcieu.ac.uk/) [22]. The outcome dataset of sleep disorders was obtained from the FinnGenR10 with 49,880 cases and 358,194 controls (https://storage.googleapis.com/finngen-public-data-r10/summary_stats/finngen_R10_SLEEP.gz) [23]. Missing data was deleted. The exposure and outcome data above are from European populations. |
|  | d) | Explain how missing data were addressed | 4 | Missing data was deleted. |
|  | e) | If applicable, indicate how multiple testing was addressed | 7 | All IVW results were corrected by multiple tests using FDR method. |
| 7 | **Assessment of assumptions** | Describe any methods or prior knowledge used to assess the assumptions or justify their validity | 6 | Cochran’s Q-statistic was used for heterogeneity test, and the Q_pvals larger than 0.05 were considered no heterogeneity. MR-Egger intercept method was performed for horizontal pleiotropy test, and the samples with p values < 0.05 were excluded from the subsequent analyses. In addition, the effect of removing each SNP on the remaining SNPS was assessed using the leave-one-out method. |
| 8 | **Sensitivity analyses and additional analyses** | Describe any sensitivity analyses or additional analyses performed (e.g. comparison of effect estimates from different approaches, independent replication, bias analytic techniques, validation of instruments, simulations) | 6 | Cochran’s Q-statistic was used for heterogeneity test, and the Q_pvals larger than 0.05 were considered no heterogeneity. MR-Egger intercept method was performed for horizontal pleiotropy test, and the samples with p values < 0.05 were excluded from the subsequent analyses. In addition, the effect of removing each SNP on the remaining SNPS was assessed using the leave-one-out method. |
| 9 | **Software and pre-registration** |  |  |  |
|  | a) | Name statistical software and package(s), including version and settings used | 7 | Statistical analyses were performed using R software (version 4.3.3). R packages ‘MRPRESSO’ [26], ‘TwoSampleMR’ [27], and ‘MendelianRandomization’ [28] were used for MR analysis. |
|  | b) | State whether the study protocol and details were pre-registered (as well as when and where) | 4 | The protocol and details of the GWAS studies included in this study were pre-registered (accession IDs GCST90092816, GCST90092817, GCST90092880, GCST90092881, GCST90092928, GCST90092931-GCST90092935, GCST90092939, GCST90092980, GCST90092987, https://www.ebi.ac.uk/gwas/). |
|  | **RESULTS** |  |  |  |
| 10 | **Descriptive data** |  |  |  |
|  | a) | Report the numbers of individuals at each stage of included studies and reasons for exclusion. Consider use of a flow diagram | 6 | Table 1 |
|  | b) | Report summary statistics for phenotypic exposure(s), outcome(s), and other relevant variables (e.g. means, SDs, proportions) | 15 | Causal associations between 13 fatty-acid-related traits and risk of sleep disorders were analyzed using 5 MR methods including IVW, MR Egger, simple mode, weighted median, and weighted mode. P values of these results were shown in Figure 4A. Based on IVW results, among the 13 fatty-acid-related traits, omega-6/total FA was found causally associated with risk of sleep disorders (IVW OR = 0.930, 95% CI: 0.880-0.983, P = 0.011; Figure 4B). The results of the weighted median and weighted mode also supported this causal relationship (weighted median OR = 0.898, 95% CI: 0.838-0.963, P = 0.003; weighted mode OR = 0.928, 95% CI: 0.866-0.993, P = 0.034; Figure 4B). The results were further displayed in a scatterplot in Figure 4C. The 5 different colored lines represent the 5 methods of MR analysis, with the slope of each line indicating the direction of the causal relationship (Figure 4C). According to the OR value and the scatterplot results, omega-6/total FA was a protective factor for sleep disorders. The funnel plot showed that the distribution of scatter points was generally symmetrical, indicating that there was no significant bias in the results of the study (Figure 4D). |
|  | c) | If the data sources include meta-analyses of previous studies, provide the assessments of heterogeneity across these studies | 16 | Table 6 |
|  | d) | For two-sample MR:  i.  Provide justification of the similarity of the genetic variant-exposure associations between the exposure and outcome samples  ii.  Provide information on the number of individuals who overlap between the exposure and outcome studies | 15 | Causal associations between 13 fatty-acid-related traits and risk of sleep disorders were analyzed using 5 MR methods including IVW, MR Egger, simple mode, weighted median, and weighted mode. P values of these results were shown in Figure 4A. Based on IVW results, among the 13 fatty-acid-related traits, omega-6/total FA was found causally associated with risk of sleep disorders (IVW OR = 0.930, 95% CI: 0.880-0.983, P = 0.011; Figure 4B). The results of the weighted median and weighted mode also supported this causal relationship (weighted median OR = 0.898, 95% CI: 0.838-0.963, P = 0.003; weighted mode OR = 0.928, 95% CI: 0.866-0.993, P = 0.034; Figure 4B). The results were further displayed in a scatterplot in Figure 4C. The 5 different colored lines represent the 5 methods of MR analysis, with the slope of each line indicating the direction of the causal relationship (Figure 4C). According to the OR value and the scatterplot results, omega-6/total FA was a protective factor for sleep disorders. The funnel plot showed that the distribution of scatter points was generally symmetrical, indicating that there was no significant bias in the results of the study (Figure 4D). |
| 11 | **Main results** |  |  |  |
|  | a) | Report the associations between genetic variant and exposure, and between genetic variant and outcome, preferably on an interpretable scale | 15 | Causal associations between 13 fatty-acid-related traits and risk of sleep disorders were analyzed using 5 MR methods including IVW, MR Egger, simple mode, weighted median, and weighted mode. P values of these results were shown in Figure 4A. Based on IVW results, among the 13 fatty-acid-related traits, omega-6/total FA was found causally associated with risk of sleep disorders (IVW OR = 0.930, 95% CI: 0.880-0.983, P = 0.011; Figure 4B). The results of the weighted median and weighted mode also supported this causal relationship (weighted median OR = 0.898, 95% CI: 0.838-0.963, P = 0.003; weighted mode OR = 0.928, 95% CI: 0.866-0.993, P = 0.034; Figure 4B). The results were further displayed in a scatterplot in Figure 4C. The 5 different colored lines represent the 5 methods of MR analysis, with the slope of each line indicating the direction of the causal relationship (Figure 4C). According to the OR value and the scatterplot results, omega-6/total FA was a protective factor for sleep disorders. The funnel plot showed that the distribution of scatter points was generally symmetrical, indicating that there was no significant bias in the results of the study (Figure 4D). |
|  | b) | Report MR estimates of the relationship between exposure and outcome, and the measures of uncertainty from the MR analysis, on an interpretable scale, such as odds ratio or relative risk per SD difference | 15 | Causal associations between 13 fatty-acid-related traits and risk of sleep disorders were analyzed using 5 MR methods including IVW, MR Egger, simple mode, weighted median, and weighted mode. P values of these results were shown in Figure 4A. Based on IVW results, among the 13 fatty-acid-related traits, omega-6/total FA was found causally associated with risk of sleep disorders (IVW OR = 0.930, 95% CI: 0.880-0.983, P = 0.011; Figure 4B). The results of the weighted median and weighted mode also supported this causal relationship (weighted median OR = 0.898, 95% CI: 0.838-0.963, P = 0.003; weighted mode OR = 0.928, 95% CI: 0.866-0.993, P = 0.034; Figure 4B). The results were further displayed in a scatterplot in Figure 4C. The 5 different colored lines represent the 5 methods of MR analysis, with the slope of each line indicating the direction of the causal relationship (Figure 4C). According to the OR value and the scatterplot results, omega-6/total FA was a protective factor for sleep disorders. The funnel plot showed that the distribution of scatter points was generally symmetrical, indicating that there was no significant bias in the results of the study (Figure 4D). |
|  | c) | If relevant, consider translating estimates of relative risk into absolute risk for a meaningful time period | NA | NA |
|  | d) | Consider plots to visualize results (e.g. forest plot, scatterplot of associations between genetic variants and outcome versus between genetic variants and exposure) | 15 | Figure 4 |
| 12 | **Assessment of assumptions** |  |  |  |
|  | a) | Report the assessment of the validity of the assumptions | 15 | Causal associations between 13 fatty-acid-related traits and risk of sleep disorders were analyzed using 5 MR methods including IVW, MR Egger, simple mode, weighted median, and weighted mode. P values of these results were shown in Figure 4A. Based on IVW results, among the 13 fatty-acid-related traits, omega-6/total FA was found causally associated with risk of sleep disorders (IVW OR = 0.930, 95% CI: 0.880-0.983, P = 0.011; Figure 4B). The results of the weighted median and weighted mode also supported this causal relationship (weighted median OR = 0.898, 95% CI: 0.838-0.963, P = 0.003; weighted mode OR = 0.928, 95% CI: 0.866-0.993, P = 0.034; Figure 4B). The results were further displayed in a scatterplot in Figure 4C. The 5 different colored lines represent the 5 methods of MR analysis, with the slope of each line indicating the direction of the causal relationship (Figure 4C). According to the OR value and the scatterplot results, omega-6/total FA was a protective factor for sleep disorders. The funnel plot showed that the distribution of scatter points was generally symmetrical, indicating that there was no significant bias in the results of the study (Figure 4D). |
|  | b) | Report any additional statistics (e.g., assessments of heterogeneity across genetic variants, such as *I^2^*, Q statistic or E-value) | 16 | Table 6 |
| 13 | **Sensitivity analyses and additional analyses** |  |  |  |
|  | a) | Report any sensitivity analyses to assess the robustness of the main results to violations of the assumptions | 16 | Table 6 |
|  | b) | Report results from other sensitivity analyses or additional analyses | 16 | In addition, the leave-one-out analysis showed that the removal of any single SNPs did not have an impact on the results (Figure S1). |
|  | c) | Report any assessment of direction of causal relationship (e.g., bidirectional MR) | NA | NA |
|  | d) | When relevant, report and compare with estimates from non-MR analyses | 18 | Nevertheless, the function of ω-6 FA in sleep remains uncertain. Some studies have proposed that ω-6 FA may have a distinct role in sleep modulation compared to ω-3 FA [13]. Our findings contrast with those of some previous studies, which have suggested a positive association between ω-6 intake/levels and sleep disorders [14, 46]. This discrepancy may be attributable to differences in dietary patterns, genetic factors, or the specific types of ω-6 FA consumed, and further investigation is warranted. |
|  | e) | Consider additional plots to visualize results (e.g., leave-one-out analyses) | 16 | In addition, the leave-one-out analysis showed that the removal of any single SNPs did not have an impact on the results (Figure S1). |
|  | **DISCUSSION** |  |  |  |
| 14 | **Key results** | Summarize key results with reference to study objectives | 16 | The current study, leveraging data from the National Health and Nutrition Examination Survey (NHANES) from 2005 to 2018, provided a comprehensive examination of the relationship between omega-3 (ω-3) and omega-6 (ω-6) polyunsaturated fatty acid intake and sleep disorders among US adults. Our findings revealed that participants with higher ω-3 FA intake had a reduced risk of sleep disorders, aligning with a growing body of literature that suggested a beneficial role of ω-3 FA in sleep health [9, 10]. However, our Mendelian randomization (MR) analysis indicated that a higher ratio of ω-6 to total fatty acid levels was causally associated with a lower risk of sleep disorders (IVW OR = 0.930, 95% CI: 0.880-0.983, P = 0.011), a novel finding that adds depth to our understanding of the role of dietary fats in sleep modulation [20]. The results yielded a significant contribution to the field, given the inconsistent results from observational studies [3]. |
| 15 | **Limitations** | Discuss limitations of the study, taking into account the validity of the IV assumptions, other sources of potential bias, and imprecision. Discuss both direction and magnitude of any potential bias and any efforts to address them | 18-19 | While the study has several strengths, it is important to acknowledge the limitations of the research. The cross-sectional nature of the NHANES data precludes the establishment of causality and the inference of temporality in the relationship between PUFA intake and sleep disorders. Furthermore, dietary intake was evaluated through self-reported 24-hour dietary recalls, which may be susceptible to recall bias and may not accurately reflect long-term dietary habits [24]. Additionally, the generalizability of our findings may be limited by the fact that our study population is restricted to non-institutionalized US civilians. Furthermore, the accuracy of the data is affected by recall bias, which represents a common issue in nutritional epidemiology. This is an inherent limitation of studies that rely on self-reported dietary intake data, and our study is no exception. The use of objective measures of dietary intake, such as biomarkers, in future studies could help to address this limitation [25]. A further limitation is the potential for unmeasured confounding. Despite the adjustment for a comprehensive range of potential confounding variables, it is possible that other factors influencing both PUFA intake and sleep disorders were not fully accounted for in the analysis. For example, mental health conditions, such as depression and anxiety, have been demonstrated to impact both sleep and dietary habits. However, these factors were not fully captured in our study [11]. In addition, it should be noted that the outcome measure of sleep disorders was based on self-reported data, which may not be as accurate as clinical diagnoses. Future studies employing objective measures of sleep, such as polysomnography, may yield more precise estimates of the relationship between PUFA intake and sleep disorders [5]. |
| 16 | **Interpretation** |  |  |  |
|  | a) | Meaning: Give a cautious overall interpretation of results in the context of their limitations and in comparison with other studies | 17-18 | Sleep is an indispensable physiological phenomenon for humans and is influenced by a variety of environmental factors and life-history traits [29, 30]. The relationship between polyunsaturated fatty acid (PUFA) intake and sleep has been the subject of extensive research, with findings that have been inconsistent. The ω-3 FA family, consisting mainly of alpha-linolenic acid, eicosapentaenoic acid (EPA) and docosahexaenoic acid (DHA), are known for their anti-inﬂammatory and anti-oxidative properties [31-33]. Several animal studies revealed that ω-3 FA may be associated with sleep onset and maintenance by regulating melatonin composition and neuronal membrane structure [34-37]. While some studies have reported a positive association between ω-3 FA levels and human sleep quality [9, 12], others have found no association or even a negative association [18, 19, 38]. For instance, a cross-sectional study employing bootstrap and rolling-window analyses by Liu et al. demonstrated that DHA intake was associated with improved sleep duration among women aged >44 years [39]. The discrepancies between their findings and ours may stem from several key factors. First, the studies assessed distinct sleep-related outcomes: while our investigation examined general sleep disorders, Liu et al. specifically focused on very short sleep duration (<5 hours). Second, the study populations differed substantially—our analysis encompassed a broad adult cohort, whereas Liu et al. conducted age- and sex-stratified analyses, uncovering more nuanced associations. Third, methodological differences likely contributed to divergent results; notably, our Mendelian randomization approach contrasts with their rolling-window and bootstrap techniques. Supporting evidence from randomized controlled trials (RCTs) suggested that DHA supplementation enhances sleep quality in healthy adults [40-42]. In contrast, interventions rich in EPA showed no significant effects in specific subgroups, such as menopausal women and migraine sufferers [42]. Furthermore, Gangitano et al. synthesized evidence indicating that high-fat diets—particularly those rich in saturated fats—may adversely affect sleep quality by inducing circadian desynchrony and disrupting metabolic and clock-related gene expression [43]. However, the effects of unsaturated fatty acids (e.g., ω-3 and ω-6 PUFAs) on sleep architecture remain inconclusive [43]. The findings of our study align more closely with those of the former group, indicating a beneficial role of ω-3 FA in sleep health. Overall, the discrepancy in these findings may be attributed to variations in study design, population characteristics, and the methods used to assess sleep disorders. Our use of Mendelian randomization (MR) analysis to evaluate causal effects represents a significant methodological advancement over simpler observational studies [44]. A recent MR study by Zuo et al. explored the causal relationships among insomnia, circulating fatty acids, and heart failure [45]. Their findings revealed that genetically predicted insomnia significantly elevated the risk of heart failure, with fatty acids—particularly saturated and monounsaturated types—mediating this association. Interestingly, while higher ω-3 levels were inversely correlated with insomnia, they exhibited a positive association with heart failure risk [45]. These results contrast with our MR analysis, which identified a protective effect of a higher ω-6 to total fatty acid ratio against sleep disorders. The discrepancies between the two studies may stem from several factors. Firstly, the outcome measure in their study was insomnia, while our study focused on a broader concept of sleep disorders, which may include various types of sleep-related problems. Different definitions and measurements of the outcome can lead to different results. Secondly, unmeasured confounders, such as mental health status in sleep research or metabolic dysfunction in cardiovascular studies, could differentially bias the observed associations. Thirdly, the scope of fatty acids examined varies: Zuo et al. assessed a broad spectrum of fatty acids within a complex cardiometabolic pathway, whereas our study specifically investigated polyunsaturated fatty acids (PUFAs) and their ratios in relation to sleep health. |
|  | b) | Mechanism: Discuss underlying biological mechanisms that could drive a potential causal relationship between the investigated exposure and the outcome, and whether the gene-environment equivalence assumption is reasonable. Use causal language carefully, clarifying that IV estimates may provide causal effects only under certain assumptions | NA | NA |
|  | c) | Clinical relevance: Discuss whether the results have clinical or public policy relevance, and to what extent they inform effect sizes of possible interventions | 19 | In conclusion, our study provides novel insights into the relationship between PUFA intake and sleep disorders, indicating the potential benefits of dietary modification for sleep health. Although our findings are encouraging, further research is required to ascertain causality and to develop evidence-based dietary recommendations for the prevention and treatment of sleep disorders. |
| 17 | **Generalizability** | Discuss the generalizability of the study results (a) to other populations, (b) across other exposure periods/timings, and (c) across other levels of exposure | 18-19 | The cross-sectional nature of the NHANES data precludes the establishment of causality and the inference of temporality in the relationship between PUFA intake and sleep disorders. Furthermore, dietary intake was evaluated through self-reported 24-hour dietary recalls, which may be susceptible to recall bias and may not accurately reflect long-term dietary habits [24]. Additionally, the generalizability of our findings may be limited by the fact that our study population is restricted to non-institutionalized US civilians. Furthermore, the accuracy of the data is affected by recall bias, which represents a common issue in nutritional epidemiology. This is an inherent limitation of studies that rely on self-reported dietary intake data, and our study is no exception. The use of objective measures of dietary intake, such as biomarkers, in future studies could help to address this limitation [25]. A further limitation is the potential for unmeasured confounding. Despite the adjustment for a comprehensive range of potential confounding variables, it is possible that other factors influencing both PUFA intake and sleep disorders were not fully accounted for in the analysis. For example, mental health conditions, such as depression and anxiety, have been demonstrated to impact both sleep and dietary habits. However, these factors were not fully captured in our study [11]. In addition, it should be noted that the outcome measure of sleep disorders was based on self-reported data, which may not be as accurate as clinical diagnoses. Future studies employing objective measures of sleep, such as polysomnography, may yield more precise estimates of the relationship between PUFA intake and sleep disorders [5]. |
|  | **OTHER INFORMATION** |  |  |  |
| 18 | **Funding** | Describe sources of funding and the role of funders in the present study and, if applicable, sources of funding for the databases and original study or studies on which the present study is based | 20 | This study was supported by the Jilin Science and Technology Department Project (20240305084YY), the National Natural Science Foundation of China [82203647], and Free Exploration Project of Natural Science Foundation of Jilin Province [YDZJ202501ZYTS728]. |
| 19 | **Data and data sharing** | Provide the data used to perform all analyses or report where and how the data can be accessed, and reference these sources in the article. Provide the statistical code needed to reproduce the results in the article, or report whether the code is publicly accessible and if so, where | 4 | The datasets for fatty-acid-related traits, including omega-3 FA, omega-6 FA, omega-6/omega-3, omega-3/total FA, omega-6/total FA, docosahexaenoic acid, docosahexaenoic acid/total FA, linoleic acid, linoleic acid/total FA, monounsaturated FA, polyunsaturated FA, saturated FA, and total FA, were obtained from the MRCIEU GWAS (https://gwas.mrcieu.ac.uk/) [22]. The outcome dataset of sleep disorders was obtained from the FinnGenR10 with 49,880 cases and 358,194 controls (https://storage.googleapis.com/finngen-public-data-r10/summary_stats/finngen_R10_SLEEP.gz) [23]. |
| 20 | **Conflicts of Interest** | All authors should declare all potential conflicts of interest | 20 | The authors declare no conflict of interest in this study. |

This checklist is copyrighted by the Equator Network under the Creative Commons Attribution 3.0 Unported (CC BY 3.0) license.

1. Skrivankova VW, Richmond RC, Woolf BAR, Yarmolinsky J, Davies NM, Swanson SA, et al. Strengthening the Reporting of Observational Studies in Epidemiology using Mendelian Randomization (STROBE-MR) Statement. JAMA. 2021;under review.

2. Skrivankova VW, Richmond RC, Woolf BAR, Davies NM, Swanson SA, VanderWeele TJ, et al. Strengthening the Reporting of Observational Studies in Epidemiology using Mendelian Randomisation (STROBE-MR): Explanation and Elaboration. BMJ. 2021;375:n2233.
